# Structural determinants explain caries differences among preschool children in Chile’s Metropolitan Region

**DOI:** 10.1186/s12903-023-02778-6

**Published:** 2023-03-09

**Authors:** María José Monsalves, Iris Espinoza, Patricia Moya, Josefina Aubert, Doris Durán, Oscar Arteaga, Jay S. Kaufman, Shrikant I. Bangdiwala

**Affiliations:** 1 Facultad de Medicina y Ciencia, Universidad San Sebastián, Lota 2465, 7510157 Santiago, Chile; 2 Departamento de Patología y Medicina Oral y Centro de Epidemiología y Vigilancia de Enfermedades Orales (CEVEO), Facultad de Odontología, Universidad de Chile, 380544 Santiago, Chile; 3 Facultad de Odontología, Universidad Finis Terrae, 7501015 Santiago, Chile; 4 Instituto de Investigación en Ciencias Odontológicas (ICOD), Facultad de Odontología, Universidad de Chile, 380544 Santiago, Chile; 5 Department of Epidemiology, Biostatistics and Occupational Health, McGill University, Montreal, QC H3A1G1 Canada; 6 Escuela de Salud Pública, Facultad de Medicina, Universidad de Chile, 8380453 Santiago, Chile; 7 Population Health Research Institute, McMaster University, Hamilton, ON L8L2X2 Canada; 8 Department of Health Research Methods, Evidence, and Impact, McMaster University, Hamilton, ON L8N 3Z5 Canada

**Keywords:** Social determinants, Inequalities, Caries, Risk factors, Children

## Abstract

**Objective:**

To estimate the association between Social Determinants of Health (structural and intermediate) and caries indicators in Chile's Metropolitan Region preschool children.

**Methods:**

A multilevel cross-sectional study of Social Determinants of Health (SDH) and caries in children aged 1 to 6 years in Chile's Metropolitan Region was conducted in 2014–2015, with three levels: district, school and child. Caries were assessed by the dmft-index and the prevalence of untreated caries. The structural determinants analyzed were Community Human Development Index (CHDI), urban/rural location, school type, caregiver's education and family income. Poisson multilevel regression models were fit.

**Results:**

The sample size was 2,275 children from 40 schools in 13 districts. While the highest CHDI district had an untreated caries prevalence of 17.1% (12.3–22.7%), in the most disadvantaged district it was 53.9% (95% CI 46.0–61.6%). As family income increased, the probability of untreated caries prevalence decreased (PR = 0.9 95% CI 0.8–1.0). Rural districts had an average dmft-index of 7.3 (95% CI 7.2–7.4), while in urban districts, it was 4.4 (95% CI 4.3–4.5). Higher probabilities of untreated caries prevalence (PR = 3.0 95% CI 2.3–3.9) were observed in rural children. Greater probabilities of untreated caries prevalence (PR = 1.3 95% CI 1.1–1.6) and prevalence of caries experience (PR = 1.3 95% CI 1.1–1.5) were observed in children whose caregivers had a secondary educational level.

**Conclusions:**

A strong association was observed between the social determinants of health, specifically the structural ones, and the caries indicators studied in children of the Metropolitan Region of Chile. There were notable differences in caries between districts according to social advantage. Rurality and caregiver's education were the most consistent predictors.

**Supplementary Information:**

The online version contains supplementary material available at 10.1186/s12903-023-02778-6.

## Introduction

Dental caries is an ongoing and critical public health problem worldwide, with prevalence up to 90% in children and adolescents in South America [1, 2]. The disadvantaged and socially marginalized populations carry the greatest burden [3].

The Commission on Social Determinants of Health of the World Health Organization (CSDH-WHO) has emphasized the relevance of studying structural and intermediate social determinants of health to understand the causes of the unequal distribution in population's health status and implement public policies considering them [4].

Several studies have shown a strong association between both structural and intermediate social determinants with oral health in children [3, 5–11]. Do [3] showed important variations in oral health of children from countries with different economic development, which are broader in countries where social disparities are larger. While Schwendicke et al. [9] concluded that caries experience among adolescents was distributed more disproportionately in high-income countries than low-income countries. To the above is added that Baker et al. [10] reported that 5 to 21% of the variance of oral health related quality of life scores in children was due to structural social determinants on 11 countries. Peres et al. [11] reported consistent area-level socioeconomic inequalities in dental caries in a large sample of Australian children. The latter study showed that modifiable factors explained 64.3% and 70.4% of the total area-level socioeconomic inequalities in primary and permanent children’s caries, respectively.

Most recent systematic reviews report that there is evidence of sufficient quality to establish an association between a lower level of parental education and an increase in early childhood caries in preschoolers [11–13]. However, for socioeconomic level and rurality, the evidence level is insufficient and should continue to be studied.

Despite the fact that Social Determinants of Health (SDH) studies in Latin-American children are more limited, some reports have suggested that oral health differences are strongly associated with family socioeconomic level, caregivers’ educational level, family income and access to fluoridated water [15–18]. However, the evidence is not yet conclusive, since most studies analyze the structural and intermediate social determinants separately and there are relevant differences between economic development and culture between countries in the region.

Chile is one of the most economically developed countries in the Latin America [19]; however, it is considered one of the most socioeconomically unequal in the world. The top 10% of population earns almost 30 times more than the bottom 50% [20]. Since the Health Reform of 2005, different governments have tried to improve access to dental care for groups recognized as vulnerable (children under 6 years of age and elderlies) through specialized policies and dental programs [21].

To date, the relationship between Chilean children’s oral health status and SDH is unknown. Our study aimed to estimate the association between Social Determinants of Health (structural and intermediate) and caries in preschool children in Chile’s Metropolitan Region, the most densely populated area of the country.

## Methods

This manuscript reports our study using STROBE (Strengthening the Reporting of Observational Studies in Epidemiology) guidelines [22] and the LEVEL (Logical Explanations & Visualizations of Estimates in Linear mixed models) checklist [23].

A multilevel cross-sectional study was conducted in 2014–2015 with three levels: district (administrative unit in Chile), school (preschools and kindergarten) and child (see Table 1). The target population were children aged 1 to 6 years from Chile’s Metropolitan Region. This region represents 37% of the entire national population and it is made up of 52 districts. All the districts in the Metropolitan Region were classified according to the Community Human Development Index (CHDI) in quintiles of social advantage: Very High, High, Middle, Low, and Very Low CHDI [24].

**Table 1:** Multilevel diagram for the association between structural and intermediate social determinants, and caries indicators in children of Chile’s Metropolitan Region

Sub-index for multilevel mixed model equations	Level	Variables
**I** (3)	District	CHDI (Community Human Development Index)^SSD^ Rural location^SSD^
**J** (18)	School	Type of school^SSD^ Educational programs of oral health School topical fluoride program Healthy snack kiosk available
**K** (29)	Child	Parental education level^SSD^ Family income^SSD^ Age Sex Type of health insurance Dental visit Using feeding bottle Tooth brushing frequency Dietary intake (different groups) dmft-index* Untreated Caries prevalence (UCP)*

SSD (Structural Social Determinants); *Caries indicators-outcomes

A sampling frame was created from official information of the Chilean Ministry of Education, considering all types of schools (public, private-subsidized and private-paid) in each district. Thirteen districts were randomly selected within CHDI quintiles. Then, forty schools were selected within each district, through simple random sampling using a random number generator app. All selected districts participated, but the private pre-schools of the highest CHDI district declined the invitation; thus, that district had only public pre-schools participating. Estimation of the necessary number of children and pre-schools to include considered the expected Intraclass Correlation Coefficients (ICCs) and Variance Inflation Factors (VIFs) based on national studies. All eligible children of each school were invited to participate, and refusal (by parents) was less than 1%.

Dental examinations were carried out between March 2014 and April 2015 in accordance with WHO guidelines [25]. Two clinical teams carried out dental examinations in schools, with an “almost perfect” reliability (inter-examiner kappa = 0.88) [26] for dmft-index. Additionally, we collected structural and intermediate determinants information from caregivers/parents and school managers. The caregiver’s questionnaire included: socioeconomic and educational level; main caregiver’s oral health; child’s general and oral health; child dietary intake; use of dental services and oral health behaviors. The school manager’s questionnaire assessed school contextual variables such as oral health education programs, topical fluoride actions in school, and healthy snacks kiosks. All schools included in our study had access to fluoridated drinking water and similar lunch menus, in accordance with the Chilean Ministry of Education guidelines.

All children were examined to create three dental indicators of caries: (1) Untreated caries prevalence (UCP) corresponding to decayed teeth at the time of the examination, not including white spot. (2) Prevalence of caries experience (dmft-prevalence) equal to 0 if dmft = 0, or equal to 1 if dmft ≥ 1; dmft corresponding to the sum of decayed, missing or filled deciduous teeth, by child and (3) Caries damage count (dmft ≥ 1) corresponding to a truncated positive count variable of dmft, excluding dmft equal to zero. The truncated analysis was done because the variables that explain having one more tooth with caries may be different from those that explain the possibility of ceasing to be healthy, although the unit of change mathematically in both cases is one.

### Structural social determinants of oral health (SSDH)

Five socioeconomic indicators were included as SSDH: (1) Community Human Development Index (CHDI), (2) urban/rural location of the district, (3) school type, (4) caregiver’s education, and (5) family income (see Table 1).

CHDI is a local adjustment indicator created by the Chilean office of the United Nations Development Program [24]. CHDI estimates district-level development conceptualized as social advantage in the country and allows comparison between districts. Interpretation of this indicator is national; it cannot be used to compare across countries. To define location as urban or rural, we used the Chilean National Statistics Institute (INE) criteria, which defines a rural community as less than 1,000 inhabitants, or with less than 50% of the population working in secondary or tertiary occupational activities. School type is defined by the Chilean Ministry of Education as public, private subsidized (partially subsidized by the government), and private paid (not subsidized by the government).

Formal caregiver/parents’ education level was categorized as: primary (up to 8 years), secondary (8 to 12 years), and post-secondary (more than 12 years). Family income at the examination was considered as an ordinal variable of six categories, with the lowest category at the Chilean monthly minimum salary CLP $225,000 (approximately USD $300), and each category increasing in increments of CLP $500,000 (approximately USD $700).

### Intermediate social determinants of oral health (ISDH)

Ten explanatory variables were included as ISDH: (1) educational programs of oral health (available or not), (2) school topical fluoride program (available or not), (3) healthy snacks kiosk (yes/no), (4) biological sex (boy/girl), (5) child age, (6) type of health insurance (public or private), (7) tooth brushing frequency (times a week), (8) gingivitis prevalence (inflammation and bleeding gums associated with plaque, yes/no), (9) ever having a dental visit (yes/no), (10) current use of a feeding bottle (yes/no), and (11) dietary intake. Dietary intake considered the consumption of sugar-sweetened beverages, candy, sugar-sweetened cookies or cakes, honey or jam (each categorized as several times per month, once a week, several times a week, once a day and several times a day).

### Statistical analyses

First, a descriptive analysis of the dmft-index and the untreated caries prevalence was performed, using means and standard deviations, and prevalences and 95% CI by district, respectively. Since the dmft-index presents overdispersion and excesses of zeros, in subsequent analyzes it was preferred to separate caries experience prevalence (dmft-prevalence) and caries damage count (dmft ≥ 1). Then, we fitted a multilevel regression model to incorporate cluster‐specific fixed effects at the district level and random effects for the school level, according to the data structure [23, 27]. We explored collinearity between location area, CHDI and district level, based on previous national studies reports of strong correlation between them. Untreated caries prevalence (UCP) and prevalence of caries experience (dmft prevalence) were estimated to be over 30%, so a multilevel Poisson regression model with robust variance was used instead of multilevel logistic regression [28]. Also, multilevel Poisson regression models were used for caries damage count (dmft ≥ 1). Three models were carried out for each outcome: ‘null’ (no covariates), ‘intermediate’ (some covariates), and ‘final’ (fully adjusted) models. In order to decide between using fixed effects versus random effects for district, we compared both approaches using the Hausman test (*p* < 0.01), which found fixed effects to be preferable to random effects. For each model, we used random intercepts for school level and fixed effects for district level (see Additional file 1 for details of models).

As suggested by Monsalves et al. [23] we include, as supplementary information, the differences in the residual variance of random effects models among districts and among schools within districts, between the null and the final multilevel regression models (see Additional file 1: Table S1). This table provides important information on how the variables at district level reduce the residual variance in the final model. All analyses were carried out using Stata 15.0 [29].

### Ethical issues

This study was approved by the Research Ethics Committee of the Faculty of Medicine, University of Chile, Chile. A letter of invitation was sent to each school selected in the 13 districts included in our study. Subsequently, a letter of informed consent was sent to all parents or caregivers of eligible children in the schools that agreed to participate. Finally, parents provided the signed informed consent for examination, and children provided verbal assent.

## Results

A total of 2,275 children between ages 1 and 6 years in 13 districts in the Metropolitan Region were examined. Table 2 gives an overview of the main structural social determinants, sociodemographic characteristics, and caries indicators, by district. Most preschoolers studied in urban area schools and came from families with monthly income equal to, or less than, CLP $500,000 per month, with public health insurance coverage similar to the national average. Mothers were considered as primary caregivers, and most had secondary education level.

**Table 2:** Overview of main Structural Social Determinants, sociodemographic characteristics, and caries indicators by district

*Districts*	CHDI	Location	Preschool % Public	Family income (%) < $705 .11 USD monthly	Caregiver education % Up to secondary education	Health coverage % Public	N	Age mean (SD)	Females %	dmft mean (SD)	Untreated Caries prevalence (UCP) (95%CI)
D1	0.679	Urban	71.2	86.6	74.6	82.1	229	2.2 (0.8)	55.5	1.0 (2.2)	28.4 (22.6–34.7)
D2	0.697	Urban	53.9	58.5	42.1	62.3	165	3.0 (1.1)	46.7	1.0 (2.3)	26.7 (20.1–34.1)
D3	0.697	Rural	0.00	75.2	65.1	75.3	167	4.5 (1.2)	45.5	3.2 (3.7)	53.9 (46.0–61.6)
D4	0.709	Urban	43.2	73.4	63.0	73.3	266	4.5 (0.9)	45.3	2.4 (3.3)	40.6 (34.7–46.8)
D5	0.724	Rural	28.2	84.6	65.2	84.6	227	4.8 (1.3)	48.5	2.9 (3.8)	46.3 (39.6–52.9)
D6	0.735	Rural	47.7	87.1	80.5	84.9	88	4.3 (1.1)	48.9	2.5 (3.2)	42.7 (32.7–54.2)
D7	0.743	Urban	58.5	54.9	40.6	51.4	147	2.7 (1.1)	48.3	1.5 (2.8)	35.4 (26.7–43.7)
D8	0.743	Urban	90.1	90.2	80.1	82.3	171	3.3 (1.3)	47.4	1.9 (3.1)	40.4 (32.9–48.1)
D9	0.759	Rural	100	81.7	73.8	87.4	122	3.7 (1.5)	48.4	2.6 (3.4)	52.5 (43.2–61.6)
D10	0.782	Urban	0.00	25.9	22.3	41.1	114	2.9 (1.2)	50.9	1.2 (2.8)	24.6 (16.9–33.5)
D11	0.807	Urban	30.8	46.6	31.6	62.3	117	4.4 (1.7)	64.1	1.6 (2.4)	39.3 (30.4–48.8)
D12	0.911	Urban	73.1	51.7	28.2	53.5	245	5.0 (1.0)	48.6	1.8 (2.8)	29.4 (23.8–35.5)
D13	0.949	Urban	78.8	54.9	19.2	38.5	217	4.6 (0.7)	50.7	1.1 (2.2)	17.1 (12.3–22.7)
Total	–	–	56.6	68.2	52.6	67.5	2275	3.9 (0.0)	49.5	1.9 (3.1)	35.9 (33.9–37.9)

CHDI: Community Human Development Index

Overall, the mean dmft-index was 1.9 (95% CI 1.8–2.0) and untreated caries prevalence was 36.0% (95% CI 34.0–37.9). Differences were observed as age increased. The highest difference was observed between two-year-old mean dmft-index of 0.8 (95% CI 0.6–1.1), and three-year-old mean dmft-index of 1.6 (95% CI 1.3–1.8). Rural districts were the most affected, with an average dmft-index of 7.3 (95% CI 7.2–7.4), while in urban communities it was 4.4 (95% CI 4.3–4.5) in the adjusted model.

There were important differences in caries indicators between districts, especially in UCP (Table 2). The most socially advantaged district had a UCP of 17.1% (12.3–22.7), a value lower than the average, and much lower than the one observed in the most socially advantaged district (D3: lower CHDI in rural area), which had a UCP of 53.9% (95% CI 46.0–61.6).

Table 3 shows multilevel model adjusted estimates for the association between SSDH with caries indicators. Associations were observed between the five SSDH included, and at least some of the caries indicators studied. Nonetheless, rurality was the only one that was related to the three oral health indicators analyzed. Higher probabilities of having caries were observed in children studying in rural districts compared to urban, especially of UCP (PR = 3.0 95% CI 2.3–3.9). Children whose parents had secondary or primary educational level had worst indicators of caries; however, the most consistent association was observed for UCP and dmft-prevalence (PR secondary level of UCP = 1.3 95% CI 1.1–1.6 and PR secondary level of dmft-prevalence = 1.3 95% CI 1.1–1.5).

**Table 3:** Multilevel model adjusted prevalence ratio (PR) estimates and 95% CIs for the association between social determinants of health and Untreated caries prevalence, Prevalence of caries experience and Caries damage count

Indicator	Categories	Untreated Caries prevalence (UCP)	Prevalence of caries experience (dmft-prevalence)	Caries damage count (dmft ≥ 1)
		PR (95%CI)	PR (95% CI)	PR (95% CI)
CHDI	High	0.9 (0.7–1.1)	0.9 (0.8–1.1)	0.9 (0.7–1.0)
(Ref. = Low)	Very high	1.1 (0.9–1.4)	1.0 (0.8–1.3)	0.9 (0.8–1.0)
Rural		3.0 (2.3–3.9)	1.7 (1.4–1.9)	1.3 (1.2–1.5)
Type of school	Public	1.4 (0.9–2.0)	1.6 (1.1–2.2)	1.2 (0.8–1.6)
(Ref. = Private paid)	Private subsidized	1.1 (0.8–1.7)	1.2 (0.9–1.7)	1.1 (0.8–1.6)
Family income		0.9 (0.8–0.9)	0.9 (0.9–1.0)	0.9 (0.9–1.0)
Caregiver’s educational level	Primary	1.2 (0.9–1.5)	1.2 (0.9–1.4)	1.2 (0.9–1.5)
(Ref. = Post-secondary)	Secondary	1.3 (1.1–1.6)	1.3 (1.1–1.5)	1.1 (0.9–1.4)
Health insurance	Public	1.1 (0.9–1.3)	1.2 (1.0–1.4)	1.1 (0.9–1.2)
Child’s sex	Male	1.1 (1.0–1.3)	1.1 (1.0–1.2)	1.2 (1.1–1.3)
Child’s age	2 years old	3.7 (2.2–6.3)	3.7 (2.2–6.4)	1.8 (1.2–2.6)
(Ref. = 1 year old)	3 years old	6.6 (3.9–10.9)	6.6 (3.9–11.2)	1.8 (1.3–2.5)
	4 years old	7.4 (4.5–11.9)	7.9 (4.9–12.9)	1.9 (1.4–2.7)
	5 years old	7.4 (4.5–12.1)	8.3 (5.0–13.8)	2.2 (1.5–3.2)
	6 years old	6.9 (4.3–11.3)	9.2 (5.6–15.2)	2.3 (1.6–3.3)
Tooth brushing frequency	Once a week	0.9 (0.7–1.4)	0.9 (0.6–1.3)	0.9 (0.7–1.3)
(Ref. = 5 to 7 times a week)	Twice a week	0.9 (0.8–1.2)	0.9 (0.9–1.2)	1.1 (0.9–1.4)
	Four times a week	1.1 (0.9–1.2)	1.1 (0.9–1.2)	0.9 (0.9–1.1)
Gingivitis		1.4 (1.2–1.6)	1.4 (1.2–1.5)	1.1 (1.0–1.3)
Use of bottle		0.9 (0.8–1.2)	0.9 (0.8–1.1)	0.9 (0.9–1.1)
Sugar beverages consumption	Several times a month	1.1 (0.7–1.8)	1.1 (0.7–1.6)	1.1 (0.6–1.8)
(Ref. = Never)	Once a week	1.4 (0.9–2.2)	1.2 (0.8–1.7)	1.2 (0.6–2.1)
	Several times a week	1.3 (0.8–2.0)	1.1 (0.8–1.7)	1.0 (0.6–1.7)
	Once a day	1.4 (0.8–2.2)	1.1 (0.8–1.7)	1.1 (0.6–1.9)
	Several times a day	1.4 (0.9–2.2)	1.1 (0.8–1.7)	1.1 (0.6–1.9)
Candy consumption	Several times a month	0.9 (0.8–1.2)	1.1 (0.9–1.3)	1.0 (0.8–1.3)
(Ref. = Never)	Once a week	0.9 (0.8–1.1)	1.0 (0.9–1.3)	1.1 (0.9–1.3)
	Several times a week	1.1 (0.9–1.3)	1.1 (0.9–1.3)	1.1 (0.9–1.3)
	Once a day	1.2 (1.0–1.5)	1.3 (1.1–1.5)	1.1 (0.9–1.3)
	Several times a day	1.2 (0.9–1.7)	1.2 (0.9–1.6)	1.3 (0.9–1.7)
Variance of random effect of school within district		< 0.0001	< 0.0001	< 0.0001

Community human development index (CHDI), school type, family income and health insurance type showed a social gradient, but these differences were not conclusive for all caries indicators (Table 3). There was no important association between child’s caries with toothbrushing frequency or the use of feeding bottle, which presented an inverse etiological association. Presence of gingivitis was related with worse outcomes for three caries indicators analyzed. As expected, more frequent consumption of sugar-sweetened beverages and candy were associated with worse caries indicators, although these associations were generally imprecise. Boys had worse caries indicators than girls. Two-year-old children had considerably worse caries indicators than one-year-olds, with 3.7 (95% CI 2.2–6.3) times greater UCP, 3.7 (95% CI 2.2–6.4) times more dmft-prevalence, and 1.8 (95% CI 1.2–2.6) times more caries damage count.

**Fig. 1:**
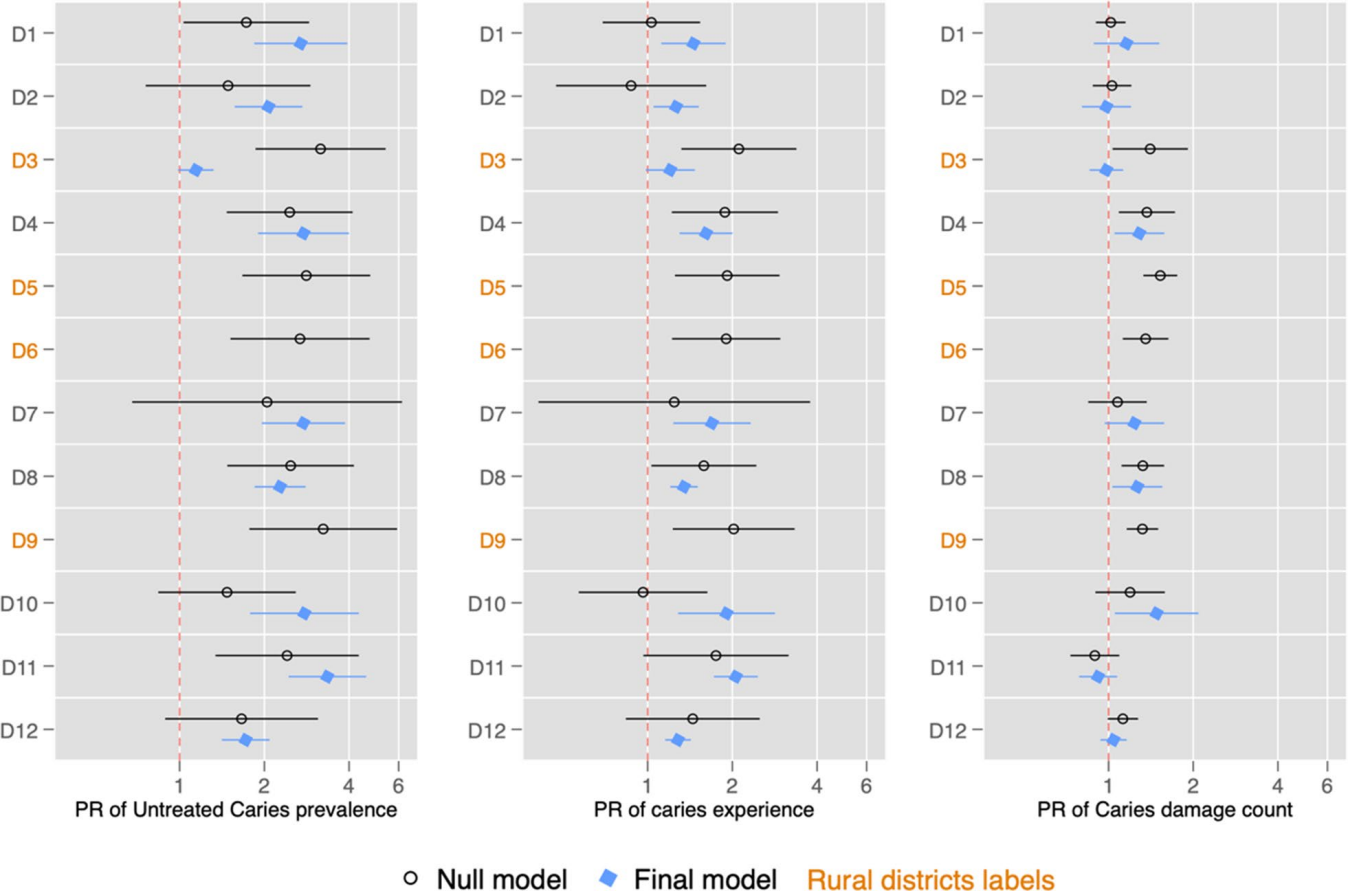
Prevalence ratio with 95% confidence intervals of null and final multilevel Poisson regression models of the district fixed effects for untreated caries prevalence, prevalence of caries experience and caries damage count, by districts (D1 to D12)

Additionally, Fig. 1 and Table 4 presents district fixed effects and the variance components for the school level from null to full multilevel models, respectively. The fixed effects at the district level show important differences between the null model and the final one. Three of the four rural districts reduce their fixed effects, since the variables included in the model explain their "district effect" very well. In urban districts, this does not occur; there is still a detectable excess risk in certain districts in relation to the reference district (D13). On the other hand, when analyzing the random effects, it is observed that the residual variance estimates are close to or equal to zero, conditioned on the predictors included. However, the variance components of the null models are not zero. This difference suggests that the covariates included in the final models substantially explain the variability in oral health indicators among schools within districts. This allows observing the effects of district level and of schools within districts, i.e. the “level effects” of oral health disparities in Chilean children. The "district effect" in urban districts cannot be explained by the components included in the final model. Rurality strongly explained the "district level effect" for UCP, dmft-prevalence and differences in caries damage count (dmft ≥ 1) among children studied.

PR = Prevalence Ratio. Some districts do not have estimates in the final model because the effect at this level was explained by the co-variables of the final model.

**Table 4:** Variance partition coefficient (VPC)* with 95% confidence intervals of between-schools within district. Null and final multilevel Poisson regression models for Untreated caries prevalence, Prevalence of caries experience and Caries damage count

Type model	VPC for untreated caries prevalence (UCP)	VPC for prevalence of caries experience (dmft-prevalence)	VPC for caries damage count (dmft ≥ 1)
Null model	0.086 (0.009–0.572)	0.089 (0.021–0.341)	0.011 (0.001–0.073)
Final model	< 0.0001	< 0.0001	< 0.0001

*VPC is the indicator of intraclass correlation in multilevel Poisson regression models

## Discussion

Our study aimed to estimate the association between social determinants of health (structural and intermediate), and untreated caries prevalence and experience (dmft-prevalence and caries damage count) in preschool children in Chile’s Metropolitan Region. Our results support the association between structural social determinants of health and the caries indicators studied in Chilean children. The social disparities, measured through the structural social determinants of health explained in large part the unequal distribution of caries among preschool children in Chile.

Globally, analyses of structural social determinants related to oral health in children are scarce, even though UCP in deciduous teeth peaks at almost 40% at age 5 [30]. To date, our study is the largest oral health examination conducted in children from the Metropolitan Region in Chile, and the first one designed for a multilevel analysis, considering child, school, and district level effects in the country. Our study has many other strengths, including good coverage of the Metropolitan Region districts, all school-types, a rate of refusal by schools of only 9% (in district D10 it was only possible to include private schools and in district D13 only public schools) and rates of refusal by parents were less than 1%. Additionally, it is the most recent study evaluating preschoolers’ health status after implementing specific policies and programs to reduce caries and associated health inequities. For example, the Explicit Health Guarantees (GES in Spanish) implemented in 2005 incorporated coverage for comprehensive oral health for 6-year-old children, focusing on prevention. More recently, the CERO program was put in place for this age group, although its implementation occurred after our study was conducted (2017).

Our study has some limitations. This study does not represent the entire reality of Chilean children, since there are many regions of Chile that have higher rates of poverty, rurality, and vulnerability than the Metropolitan Region. In addition, the limitations associated with the different schooling coverage throughout the study group should be considered. In Chile there is low school coverage in the group under four years old (between 42% in 2014 and 49% in 2021). However, this increases to more than 90% in six-year-olds [31]. Because coverage is higher in advantaged social groups, it is possible that our findings underestimate prevalence and disparities. Furthermore, some sampled private schools rejected participation in the study, and some variables were self-reported and thus subject to potential biases. The dental exams were carried out in the school-classroom, and the conditions to perform ICDAS criteria could not be guaranteed.

There are also limitations related to the caries indicators analyzed. Not cavitated caries or white spot were not considered, including them could increase the differences between the children. In addition, it is important to be careful when analyzing the differences between indicators. For example, one can define ‘caries-free children’ as those that have no UCP (untreated caries prevalence = 0) or no prevalence of caries experience (dmft-prevalence equal zero) at the moment of the examination. However, only those with no prevalence of caries experience (dmft-prevalence zero is never had caries) may be defined as “really caries-free”. This is because untreated caries prevalence obscures the history of fillings done before the measurement. Untreated caries prevalence may be determined by access to oral-health services rather than the disease itself, and in the specific case of Chile, 25.7% of the total number of people waiting for health care are waiting for oral-health services [32]. Thus, it is necessary to consider both UCP and dmft-prevalence together when addressing caries from a population perspective, in a conceptual framework that considers the health system as an intermediate determinant acting to modulate both differential exposure and differential vulnerability [4].

As expected, caries indicators studied were associated with social determinants. But poor oral health status was explained strongly and consistently by structural determinants more than by intermediate determinants. Similar studies concluded that the former are better at explaining the health differences between individuals than the latter [3, 5–18]. Do [3] explained that the differences between oral health status are best illustrated by countries’ social and macro-economic policies, while the differences within a country are defined by socioeconomic position like the Chilean situation from our findings. A systematic review of Schwendicke et al. [9] reports that there is an association between oral health status and the socioeconomic position of individuals (83 studies), where the gap is significantly greater for low educational levels in highly developed countries. This is consistent with the findings of our study. Despite advances in Chile and its more developed position in the region [19], structural determinants associated to economical position within the country continue to explain differences in health status in Chilean children.

We observed higher caries damage count (dmft ≥ 1) in preschoolers whose parents have lower educational levels, in accordance with previous international studies [6–9, 12–14]. The type of school was associated with all indicators, but more strongly with the probability of prevalence of caries experience (dmft-prevalence). Also, higher monthly family income was a protective factor in Chilean preschoolers. In Chile, these two variables are related to the socioeconomic position of children, and the inverse relationship with UCP, dmft-prevalence and caries damage count (dmft ≥ 1) are consistent with international evidence [7, 8, 16–18, 33, 34].

Districts with lower CHDI influenced the possibility of having poorer caries indicators. This association was less robust in our study than in other reports by other Latin-American authors [35–37]. However, a limitation of our study is that data for CHDI is dated 2003. Rurality was consistently associated with poor caries indicators; the stronger association was with UCP. Studies conducted in other areas of Chile, have also reported an association for rurality, even though comparison to the Metropolitan Region is not straightforward [38, 39]. Furthermore, global studies corroborate that children in rural areas tend to have poorer oral health status compared with their urban counterparts [40–42].

In relation to the intermediate social determinants of health analyzed, our findings support that age is an important variable in the possibility of having UCP (3.7 IC95% 2.2–6.3), dmft-prevalence (3.7 IC95% 2.2–6.4) and caries damage count (1.8 IC95% 1.2–2.6) from 2 years, increasing risk for each year. The greatest increase in risk of no longer being a caries-free child (UPC = 0 or dmft-prevalence = 0) was noted when a child is two years of age, settling down by the child’s 3rd birthday. This does not mean that age is more relevant than social stratification, since age-related progression in poorer oral health comes along with social disadvantage accumulation, described by some authors as embodiment [43]. From a public health perspective, with an equity focus, we must begin oral health promotion and prevention before the age of two [44–46]. Both age and sex associations found in our study agree with those from other authors [3, 47–50].

The use of feeding bottle and toothbrushing frequency were not consistently associated with the caries indicators of our study, in accordance with findings reported by other authors [1, 48]. When consumption of fermentable carbohydrates increased, all caries indicators worsened, but they do not explain the distribution of the disease at the population level. This is because the more widespread the exposure is—e.g. high sugar consumption in Chile—the less it explains the population-level distribution of the disease [51, 52].

## Conclusion

The caries differences among Chilean children were explained mainly through structural determinants. Rurality and parental education were the structural determinants most strongly associated with caries indicators, and district-level factors explained much of the variation in caries indicator disparities between districts.

These findings emphasize that the distribution of caries in a population can be a sensitive predictor of country-level disparities. Therefore, in addition to current policies for children's health, new strategies should focus on children from rural areas, addressing contextual structural determinants, and on children from urban areas addressing proximal determinants (educational level of the caregivers).

## Supplementary Information

**Additional file 1.** 1. Equations for multilevel mixed effects Poisson regression models used in the study. 2. Variance partition coefficient and 95% confidence intervals of random effects between- district and between- schools within district.

## Data Availability

The datasets used and/or analyzed during the current study are available from the first author (maria.monsalves@uss.cl) on reasonable request.
