# Supplementary material for: Structural determinants explain caries differences among preschool children in Chile’s Metropolitan Region
Source: BMC Oral Health. 2023 Mar 9;23:136. doi: 10.1186/s12903-023-02778-6 (PMC9996898; doi:10.1186/s12903-023-02778-6)
Supplement: Supplementary file 1 — Additional file 1. 1. Equations for multilevel mixed effects Poisson regression models used in the study. 2. Variance partition coefficient and 95% confidence intervals of random effects between- district and between- schools within district. [file 12903_2023_2778_MOESM1_ESM.docx]

**Supplementary Information 1**

**Equations for multilevel mixed effects Poisson regression models used for Structural determinants explain caries differences among preschool children in Chile’s Metropolitan Region**

For each outcome, we fitted 3 different multilevel Poisson regression models. First, a ‘null model’, which included only fixed effects for district and random effects for schools within districts – no independent variables. This model allows one to quantify the variation in the outcome and the proportion of this variation by level. Then, the second model, an ‘intermediate model’, included district- and school-level independent variables. Finally, we fit the ‘final model’ – with selected district-, school- and child-level independent variables according to AIC criteria and results from the intermediate model.

1. First, we fit the ‘null’ model, which included fixed effects for districts and random effects for schools within districts:

$$Y_{ijk}=log\left[ \lambda_{ijk} \right]= {[\beta}_{0}+ \beta_{0jk}] + \beta_{1k}$$

$$Y_{ijk}\sim Poisson(\lambda_{ijk})$$

(1)

where $Y_{ijk}$ is oral health outcome (Untreated caries prevalence, Prevalence of caries experience and Caries damage count) for child *i* at school *j* in district *k*. Parameter $\beta_{0jk}$ is the random effects among school-clusters within districts and $\beta_{1k}$is the fixed effect of district *k*. This model doesn’t contain any independent variables and so the purpose of this analysis is to quantify the variation in the outcome and the proportion of this variation that is due to between-schools or districts differences.

1. Second, we fit an ‘intermediate’ model that includes 15 independent variables in the model, and the district fixed effects and school‐specific random effects:

$$Y_{ijk}=log\left[ \lambda_{ijk} \right]={[\beta}_{0}+ \beta_{0jk}] + \beta_{1k} {+\beta}_{2}\mathrm{HDI}_{k}{+ \beta}_{3}{rural location}_{k}{+\beta}_{4}{type of the school}_{jk}+ \beta_{5}{school fluoride program}_{jk} {+\beta}_{6}{educational programs of oral health}_{jk}+ \beta_{7}{healthy snack kiosk}_{jk}+ \beta_{8}{parental education level}_{ijk}+ \beta_{9}{family income}_{ijk}+ \beta_{10}{age}_{ijk}+ \beta_{11}{sex}_{ijk}+ \beta_{12}{type of health insurance}_{ijk}+ \beta_{13}{dental visit}_{ijk}+ \beta_{14}{feeding bottle}_{ijk}+ \beta_{15}{toothbrushing}_{ijk}+ \beta_{16}{dietary intake}_{ijk}$$

$$Y_{ijk}\sim Poisson(\lambda_{ijk})$$

(2)

where the district-level characteristics regression coefficients are $\beta_{1-2}$ , the school-level characteristics regression coefficients are $\beta_{4-7}$ , and the child-level characteristics regression coefficients are $\beta_{8-16}$ .

1. Finally, we fit a ‘final’ model that included 11 independent variables in the model, and the district fixed effects and school‐specific random effects:

$$Y_{ijk}=log\left[ \lambda_{ijk} \right]={[\beta}_{0}+ \beta_{0jk}] + \beta_{1k} {+\beta}_{2}\mathrm{HDI}_{k}{+\beta}_{3}{rural location}_{k}{+\beta}_{4}{type of the school}_{jk}+ \beta_{5}{parental education level}_{ijk}+ \beta_{6}{family income}_{ijk}+ \beta_{7}{age}_{ijk}+ \beta_{8}{sex}_{i}jk+ \beta_{9}{type of health insurance}_{ijk}+ \beta_{10}{feeding bottle}_{ijk}+ \beta_{11}{toothbrushing}_{ijk}+ \beta_{12}{dietary intake}_{ijk}$$

$$Y_{ijk}\sim Poisson(\lambda_{ijk})$$

(3)

where the district-level characteristics regression coefficients are $\beta_{2-3}$ , the school-level characteristics regression coefficient is $\beta_{4}$ , and the child-level characteristics regression coefficients are $\beta_{5-12}$ .

In each of these 3 models, we assumed that the distribution of the random effects was Normal: $\beta_{0jk}\sim N(0,{}_{schools}^{2})$, and they are independent of the model error.

**Supplementary Information 2**

**Table S1. Variance partition coefficient and 95% confidence intervals of random effects between- district and between- schools within district. Null and final multilevel Poisson regression models**

| **Type model** | **Variance of random effect** | **Caries prevalence** | **Binary dmft** | **Truncated positive dmft** |
| --- | --- | --- | --- | --- |
| Null model | At District level | 0.07 (0.02-0.27) | 0.04 (0.01-0.27) | 0.00 |
|  | At School level within District level | 0.09 (0.03-0.23) | 0.11 (0.05-0.27) | 0.04 (0.02-0.07) |
| Final model | At District level | 0.03 (0.01-0.14) | 0.00 (0.00-0.01) | 0.00 (0.00-0.02) |
|  | At School level within District level | <0.01 (0.00-0.02) | <0.01 (0.00-0.03) | 0.01 (0.00-0.03) |
